# An empirical investigation into the preferences of the elderly for user interface design in personal electronic health record systems

**DOI:** 10.3389/fdgth.2023.1289904

**Published:** 2024-01-29

**Authors:** Sainan Zhang, Jisung Song

**Affiliations:** Graduate School of Communication Design, Hanyang University, Ansan, Republic of Korea

**Keywords:** ePHR, mobile healthcare services, interface design, conjoint analysis, elderly user requirements, health aging

## Abstract

**Background:**

With the continuous advancement of digital technologies, electronic Personal Health Records (ePHR) offer end-users greater control and convenience over their health data. Although ePHR are perceived as innovative tools in medical services that provide patient-centered care and disease prevention, many system interfaces are inclined toward younger users, overlooking investigations pertinent to elderly users. Our objective is to uncover the preferences of the elderly for an ideal ePHR system interface.

**Materials and methods:**

Relying on a literature review, we identified six interface attributes. Utilizing conjoint analysis, we constructed 16 representative design scenarios based on orthogonal design by combining different attribute levels. We invited 187 elderly participants to evaluate these scenarios. Data analysis was performed using SPSS 26.0. The results indicate that among the ePHR interface design attributes, the elderly prioritize color attributes, followed by the notification method. Designs with contrasting color schemes, skeuomorphic design approaches, and icon-centric menu navigation with segmented layouts, and voice notifications when a message is received, are the most preferred interface design choices.

**Discussion:**

This research elucidates the ideal interface design elements for ePHR as perceived by the elderly, offering valuable references for age-friendly design considerations in ePHR systems.

**Results:**

Implementing these insights can aid in promoting mobile health services among the elderly demographic, enhancing their user experience in health management interfaces. This, in turn, fosters the widespread adoption of mobile health service technologies, further advancing the development of a healthy aging society.

## 1 Introduction

The global population aged 60 and over accounts for approximately 10%, and it is projected to surpass one billion by 2030, making up about 12% of the total population (1, 2). By 2050, the population aged 60 and above in China is expected to reach 454 million, constituting 34% of the nation's total population. Rapid aging undoubtedly poses challenges to public services, with the pressure on healthcare resources escalating sharply (3). This is primarily because older individuals are more susceptible to illnesses compared to younger populations, thereby increasing their demand for healthcare resources (4), High-quality healthcare services have become essential, and the effective management of medical information is critical. Quality information has been shown to have a significant impact on healthcare outcomes (5–7). Various health departments worldwide employ electronic repositories for medical data, crafting suitable health information frameworks that securely store, exchange, and authorize user access (8, 9). Big data analytics have been widely implemented in healthcare, with developed countries leveraging Electronic Health Records (EHR) to enhance healthcare systems (10). This digital shift aims to improve the quality and efficiency of healthcare services (11). As digital substitutes for traditional paper records, EHR are gradually gaining prevalence in an increasingly digitized society. EHR offer direct management and clinical data collection capabilities that surpass their paper counterparts, often marred by errors, information gaps, and unauthorized access. These digital systems are accessible, comparable, communicable, and confidential. With the wide adoption of the Internet and healthcare information, the healthcare industry is undergoing a significant transformation. In this shift, individuals are increasingly taking an active role in their healthcare, evolving into the primary stakeholders in their health management. Expanding disease management services to non-patient demographics, valuable insights can be derived from combining data from healthy users with those with health issues (12, 13). This was particularly evident during the COVID-19 pandemic, where digital capabilities played a pivotal role in controlling disease progression.

Multifaceted issues in Human-Computer Interaction (HCI) design can lead to suboptimal utilization of ePHR. For instance, cluttered interface design and fragmented information make it challenging for users to quickly comprehend their personal information (14). When considering the usability of computer electronic systems, user engagement plays a key role in enhancing performance. This includes users’ cognition, guidance, input, and interaction with the system to achieve more efficient operations and outcomes (15, 16). However, a comprehensive assessment of the collaboration between “users and computers” is essential (17, 18). The rise of the HCI field emphasizes the connection between users and machines, primarily allowing participants in computer operations to exchange information. Existing system interfaces are generally designed to interact with a wide array of public users (19). But many systems have not been able to bring about a significant improvement in healthcare services due to issues such as poor usability or inadequate management (20, 21). Moreover, the role of specific populations among the target users is often overlooked when evaluating and redesigning processes and digital systems (22), In particular, to date research on the preferences of elderly populations for ideal ePHR interfaces is insufficient. Understanding these preferences is critical for the effective design and development of healthcare information systems targeted at seniors. Therefore, this study aims to elucidate the preferences of elderly individuals for ePHR interface design through conjoint analysis. We anticipate that the findings of our research will offer new insights into mobile health information, benefitting designers and developers in establishing settings for the interfaces of mobile health products intended for elderly users.

## 2 Literature review

An excellent design system can foster a positive sense of accomplishment in users and allow for greater focus (23), proposed guiding principles for user interface design, especially considering that the mobile health domain often involves the presentation of complex data information. Therefore, design developers should understand the diverse needs of different users. This study applies the principles from Schneiderman et al. to the mobile health medical field, with these elements aiding in functionality interpretation and emphasizing design elements that reduce understanding difficulty (23). When developing user interfaces, developers should deeply grasp the diverse needs of system users, ensuring that the interface design minimizes user operational errors. His summarized interface design guidelines include: maintaining interface consistency; providing shortcuts for regular users; giving real-time feedback on user operations; offering concise error handling; ensuring operations are reversible for user corrections; allowing users to always feel in control of operations; and alleviating the strain on users' short-term memory. A well-designed computer interface can provide users with positive feedback, thereby allowing them to focus more on their primary tasks (15). Our research suggests that the preferences of the elderly for ePHR are influenced by interface design elements that not only augment functionality but also bolster user confidence and enhance usability. For instance, Yang et al. employed conjoint analysis in their study of the design methods for smart refrigerator human-machine interfaces, analyzing user preferences for these interfaces (24) Moreover, research by Plegge et al. revealed public preferences for electronic services, identifying the key factors for the success of these services (25). In the healthcare sector, researchers have already used conjoint analysis to study patient preferences for medical services (26, 27). Li et al. utilized conjoint analysis to design personalized persuasive game elements for elderly users in health applications, which will be incorporated into the application design (28). However, there have been few studies comparing elderly preferences for interface design elements in ePHR systems. Therefore, this study aims to fill that gap.

Currently, numerous countries have adopted computer-based healthcare information management, leading to a growing reliance on information technology within the healthcare sector (29). However, one limitation of EHRs is their dependence solely on data from healthcare institutions or government reports (30). The future points towards a partial shift from institution-centric to individual-centric information management, where Personal Electronic Health Records (EHR) (31). Emerge as an extension of EHR with the goal of electronic health empowerment. This shift aims to enhance individual awareness of key health metrics in daily life (32) ePHR originates from EHR (32, 33), aiming to enhance individuals' understanding of their physiological metrics in daily life. ePHR (32) is managed by the individuals themselves. Pagliari et al. (34) summarized its seven primary functions as access to electronic clinical records from healthcare providers. Personal health records or diaries Self-management support. Facilitating communication between patients and providers. Providing links to information related to diseases, treatments, or self-care. Offering links to support resources. Gathering symptom data or health behavior data reported through electronic devices or objective monitoring. Overall, ePHR have the potential to improve the quality of care, strengthen the relationship between individuals and healthcare institutions, and reduce healthcare costs (35, 36) Proactive liberation of Electronic Medical Record (EMR) data will eventually lead to the individualized consolidation of fragmented healthcare data into personalized electronic portfolios spanning one's health ecosystem. Features like long-term health tracking in ePHR are particularly beneficial for managing chronic conditions in the elderly (7). Although ePHR systems are perceived as valuable tools, their usability can be challenging for many older adults (37). Older adults, generally conservative in adopting new technologies (38), have shown increasing acceptance and proficiency in using internet and digital products in recent years (39, 40). However, if the operational cost is high, hesitancy may arise in initial usage (41). Therefore, ensuring the usability of ePHR for older adults is a key issue in facilitating their adoption and effective use. In recent years, the need for technological breakthroughs has greatly impacted healthcare services in many developing countries. Individuals can store, manage, and access health-related information on digital platforms (42), including medical history, drug prescriptions, diagnostic results, lab reports, clinical notes, and vaccination records. Moreover, multiple studies have demonstrated the successful application of mobile health among elderly users. They confirm that ePHR can improve medication adherence among adults (43), and facilitate self-management of chronic conditions. This allows individuals to better understand and manage their health while also enabling healthcare professionals to provide improved care (44, 45) ePHR enables individuals to consolidate their health data in one place, facilitating more comprehensive medical decision-making by enabling data sharing with healthcare institutions, doctors, and other professionals. Within traditional healthcare systems, users may have to provide the same health information multiple times across different institutions; ePHR can reduce such redundancy. For elderly users, consolidated storage of personal health information, including medical history, medication records, and allergy information, aids in maintaining a comprehensive health record and reduces the risk of information loss and confusion. Sharing medical records with doctors, nurses, and other healthcare professionals facilitates better-coordinated care and more accurate diagnosis and treatment (46). In emergencies, medical personnel can access this information more quickly, enabling more accurate medical decisions, Synchronization with health indicators like blood pressure and blood sugar, along with daily activity levels, helps the elderly better manage their health (47). Online appointment scheduling, communication with doctors, and even remote diagnosis save time and energy, making ePHR an indispensable part of the modern healthcare system, continually driving the digitization of the medical industry.

Considering the pronounced interdisciplinary nature of interfaces, which also overlaps with other domains (48). Building upon the study by Kim et al. has been demonstrated that the joint analysis method is dedicated to evaluating user experience, understanding user needs, and prioritizing the development of experience-driven products (49). Our study identifies six attributes closely related to PHR user interfaces.

The first attribute, Platform Device Type, encompasses the broad use of Information Communication Technology (ICT) platforms that have profoundly transformed the healthcare sector (50). Smartphones are becoming mainstream communication tools in healthcare environments (51), and have been proven beneficial for tracking and evaluating individual health levels (52). The use of mobile devices such as smartphones and tablets is crucial, as these devices serve as primary mediums for accessing information and services required for various daily tasks (53). It is anticipated that an increasing number of elderly individuals will utilize mobile devices to meet some of their daily needs (54).

Secondly, color settings in the ePHR interfaces present a broad and complex topic that intersects nearly all aspects of human activity. References to color in art, philosophy, psychology, and science lack uniformity because they are shaped by their respective disciplines, philosophies, theories, and practices (55). In graphic design, color schemes are produced based on the corresponding positions of colors on the color wheel. These schemes can influence and emphasize objects; uniform tones have a calming effect, adjacent hues are harmonious, and complementary colors offer strong contrast (56).

The third attribute pertains to design style and the visual categorization of content within the interface, which involves a combination of object size, distance, and color (57). This directly influences users' comprehension and manipulation of the interface (58), and improper design can increase the likelihood of usage errors. Icons serve as crucial graphical elements in the interface with potent communicative potential, reflecting not only the design style but also affecting interaction quality and user experience (58). Companies like Google, Microsoft, and IBM currently employ minimalist flat designs in their operating systems, whereas Apple's Big Sur follows the trend of light skeuomorphism. Research indicates that simple designs can enhance pleasure and increase usability Such designs are highly recommended for the majority of medical decision-making systems (59). However, Cho et al, have shown that skeuomorphic designs tend to be favored by the elderly as they find them to be more comprehensible and user friendly (60).

Fourthly, for the most effective interaction between users and the system, interface layouts should provide effective visual guidance for the users. For instance, electronic personal health records designed for patients with chronic heart failure have demonstrated how to effectively distribute a range of elements, including 225 data elements, required text fields, narratives, and tracking functionalities (61). Research has also shown different layouts serving as digital calendar references, confirming the interrelations between information and tasks (62). Centrally aligned and grid-based layouts become more manageable when information is categorized into blocks.

The fifth attribute, essential for user engagement, revolves around menu navigation—an indispensable pathway for information retrieval, acting as the initial point of interaction with the system. Modern interfaces predominantly utilize icons or text within the navigation menu, processed diversely in users' cognitive realms. Previous studies have robustly advocated for the incorporation of icons within graphical user interfaces, attributing to their ability to demystify the interface's complexity and bolster information transmission efficiency. Graphic symbols stand out for their capacity to expedite and enhance the accuracy of information identification, in contrast to their textual counterparts (23). Contrarily, some scholars posit that interfaces amalgamating graphics and text manifest superior usability, optimizing task completion times and precision compared to interfaces relying solely on text or graphics (63).

Lastly, the mode of notification is another factor to consider. Designing appropriate notification mechanisms is an essential component of providing a good user experience and serves as one means of conveying information, events, or state changes to the user. Notifications can be employed in various scenarios, such as alerting users to critical information, and task reminders. They assist users in obtaining timely information, making decisions, and staying connected with the system. Popup notification boxes are prominent message containers, typically located at the top or center of the screen, used for displaying vital information, alerting the user, or requesting user actions. Audio and visual cues can aid users in comprehending the information. Users typically need to click a confirmation or close button to handle the notification or can receive messages through voice broadcasting.

## 3 Methods

### 3.1 Research design

Conjoint analysis is used to collect and analyze customer preferences and apply them to optimal product design (64). It was originally used to decompose overall evaluations and classify basic measurements of target objects. It allows for multi-faceted evaluations of virtual products, quantifying each attribute and its weight in the overall decision. Compared to other methods, conjoint analysis provides a more accurate estimate, and it can more precisely determine which individual surveys among multiple key attributes are more important (65, 66). It explores consumers' preferences and decision-making methods regarding the value of products or services. It can evaluate the relative importance of multiple elements in a product by aggregating the utility scores of each product attribute to better predict overall user preferences.

Conjoint analysis typically involves the following steps: The first step is to determine the key attributes of a product or service. Then, based on the determined product attributes and attribute levels, combinations are made using orthogonal design methods to generate a series of virtual products. Next is data collection and evaluation: invite respondents to evaluate these virtual products, using methods such as scoring and ranking, to understand their preferences for different product configurations. Finally, calculate attribute utility values: based on the evaluation by respondents, extract the utility values for each attribute and its levels, representing consumers' relative preferences for each attribute. Product preference prediction: Using the obtained utility values, predict consumer preferences among various product configurations, providing references for product design and marketing strategies (67).

Given this, due to the diversity of medical interface content and the complexity of interface interaction, compared to other methods, using CA to guide the relative importance assessment of ePHR interface design elements is necessary. However, most conjoint tests rely on fractional factorial designs, most commonly using orthogonal arrays, assuming that most interactions between independent variables only consider main effects (68). Since the combination of all factor levels might be too large for respondents to rank, a factorial design is performed.

### 3.2 Participants

We used purposeful sampling for the survey. The inclusion criteria for the sample were elderly people who can use electronic devices. Beforehand, we asked them if they were willing to participate in this research. The data was collected through an online survey, which was voluntary and uncompensated. The respondents signed an informed consent online. The researchers promised that the data would only be used for academic research. In total, 187 elderly people agreed to participate. The sample size meets the requirements of similar research (69).

### 3.3 Orthogonal design

Based on the literature review, attributes suitable for ePHR interfaces and their respective levels were identified, as shown in Table 1. Each attribute level was selected based on the effectiveness and applicability of the ePHR interface, incorporating six key elements in a 2 × 3 × 2 × 2 × 2 × 2 design, which could generate 96 possible combinations. However, it is challenging for test participants to choose among these 96 combinations, potentially leading to high variability in results. To address this issue, an orthogonal experimental design was executed using SPSS 26, optimizing 16 typical combinations, as shown in Table 2.

**Table 1:** Interface attribute factors.

Attribute	Levels description	Concept	Theoretical refs.
Platform	Smartphone	Multifunctional mobile phones equipped with computational capabilities and internet connectivity (70)	ICT in healthcare encompasses an extensive array of technological platforms (71)
	Tablet	Primarily utilizes a touchscreen interface for input, foregoing physical keyboards and mice (72)	
Color scheme	Monochromatic	Color schemes composed of hues with varying degrees of saturation and luminosity (73)	How colors are distributed can significantly impact perception, behavior, and responses (74–76)
	Analogous	Color schemes consisting of three adjacent hues on the color wheel (77)	
	Contrast	Colors that are complementary on the color wheel, are often used in design to achieve a striking contrast (77)	
Design style	Skeuomorphic	Integration of physical interface elements with contemporary digital design (78, 79)	The design of electronic health records contributes to optimal support provision for the elderly population (80)
	Flat	Design featuring minimalistic or no detail and color graduations, often employing simple solid colors for the user interface (78)	
Layout	Segmented Grid Layout	Content organized into sections with headings, breaking from the conventional grid layout commonly used in design	Implementation of access control options while enforcing distinctions between hidden and visible content (81)
	Non-conventional Grid Layout	Departure from the conventional grid-based organizational structure in layout design.	
Menu	Text-centric	Emphasis on the sections containing textual content (82)	Icons serve a pivotal role in message transmission between software and users, though they can sometimes be ambiguous in conveying intended meanings (83)
	Icon-centric	Design primarily emphasizes graphical icons (84)	
Notification	Voice	Communication of new messages or events via voice-based modalities (85)	Feedback cues hold a crucial function in system and interaction design, assisting users in comprehending their actions and the system's responses (86)
	Image	Conveyance of messages through visual imagery (87)	

**Table 2:** Orthogonal design combinations.

Card	Experimental factors					
	Platform	Color	Design	Layout	Menu	Notification
1	Tablet	Monochromatic	Flat	Chunked	Text-centered	Image
2	Tablet	Analogous	Flat	Unconventional grids	Icon-centered	Voice
3	Tablet	Contrast	Skeuomorphic	Unconventional grids	Icon-centered	Image
4	Tablet	Analogous	Skeuomorphic	Chunked	Icon-centered	Image
5	Smartphone	Analogous	Skeuomorphic	Chunked	Text-centered	Voice
6	Tablet	Monochromatic	Skeuomorphic	Chunked	Text-centered	Voice
7	Smartphone	Monochromatic	Skeuomorphic	Unconventional grids	Icon-centered	Image
8	Smartphone	Analogous	Flat	Unconventional grids	Text-centered	Image
9	Tablet	Contrast	Flat	Chunked	Icon-centered	Voice
10	Smartphone	Monochromatic	Skeuomorphic	Chunked	Icon-centered	Image
11	Tablet	Monochromatic	Skeuomorphic	Unconventional grids	Text-centered	Voice
12	Smartphone	Contrast	Flat	Unconventional grids	Text-centered	Image
13	Smartphone	Contrast	Skeuomorphic	Chunked	Icon-centered	Voice
14	Smartphone	Monochromatic	Flat	Chunked	Icon-centered	Voice
15	Tablet	Monochromatic	Flat	Unconventional grids	Text-centered	Image
16	Smartphone	Monochromatic	Flat	Unconventional grids	Icon-centered	Voice

### 3.4 Questionnaire design

Upon structuring the orthogonal schemes, the cards are meticulously organized and assessed during the questionnaire design process, aiming to culminate in conclusive results through a refined conjoint analysis. The structured survey questionnaire is bifurcated into two predominant sections. The inaugural section collates essential demographic information from respondents, encapsulating aspects such as gender, age, and educational background.

**Figure 1:**
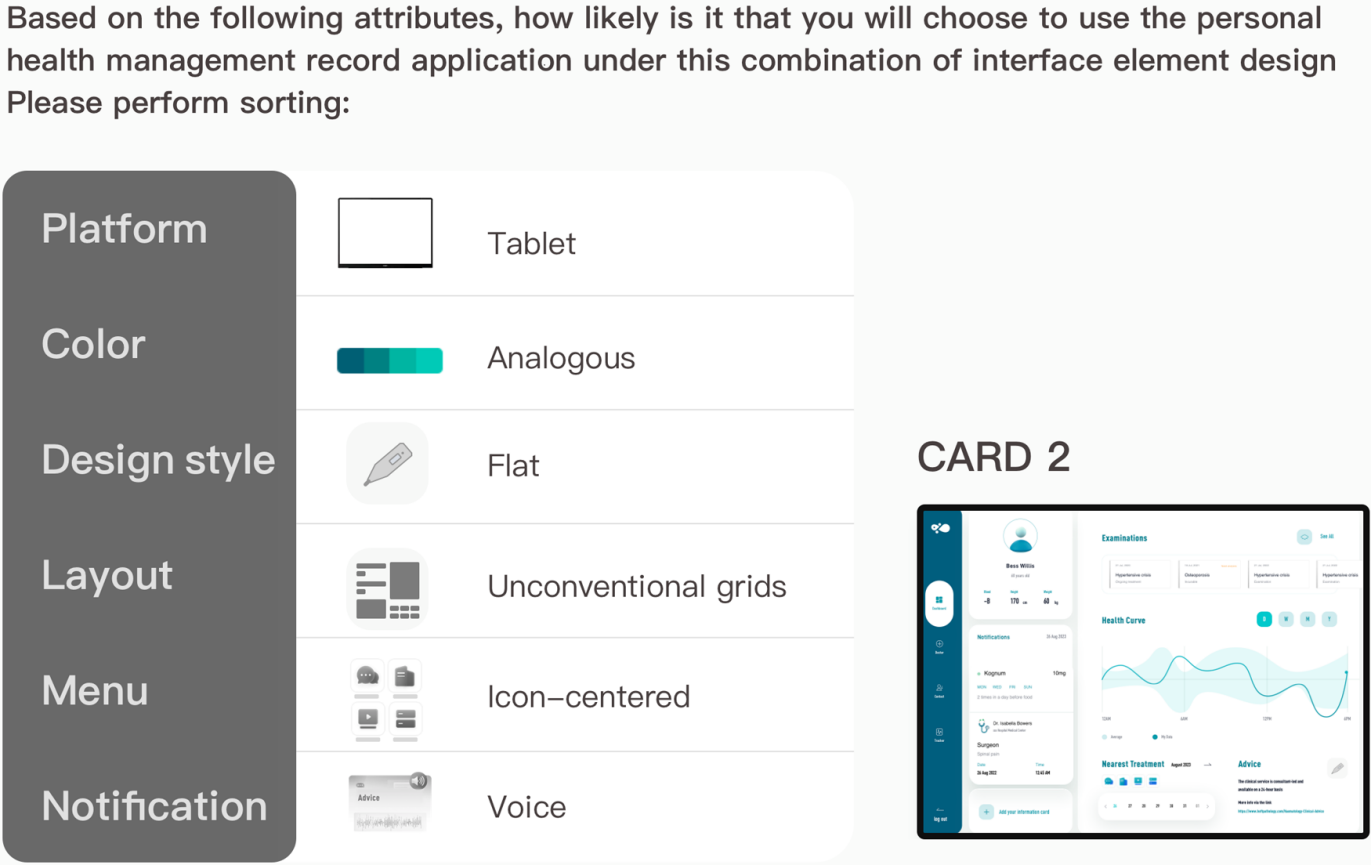
Example cards from the questionnaire.

Transitioning to the second segment, respondents are prompted to allocate rankings to diverse, numerically assorted combination schemes. An introductory overview of the questionnaire's contextual background is provided, accompanied by detailed elucidations of each constituent element. Respondents are tasked with ranking the schematic cards on a scale of 1 to 16, where a ranking of 1 signifies utmost preference, cascading down to 16, indicative of minimal preference. In this segment, the card schemes are vividly presented to respondents, epitomized in Figure 1. To encapsulate the user interface with utmost precision and clarity, high-fidelity mockups were meticulously crafted, utilizing the advanced capabilities of Sketch 83.1 software.

### 3.5 Data collection

Data were collected between August 1st and August 25th, 2023, in China, and were not restricted to a single urban area. A total of 187 elderly individuals with experience in using smart devices participated in the survey. They evaluated their preference levels for the 16 optimized typical combinations. To ensure a broad range of survey participation, the sample included respondents from diverse educational backgrounds, genders, age groups, and varying frequencies of mobile health system usage.

## 4 Results

A total of 187 questionnaires were distributed and all were returned, resulting in a 100% effective response rate. Of the 187 respondents, 119 were male (63.64%) and 68 were female (36.36%). The majority of respondents were aged between 56 and 60, accounting for 69.52% of the survey population. Half of the respondents had received at least high school-level education, and a majority indicated that they had experience using mobile health systems, as shown in Table 3.

**Table 3:** Demographic profile of respondents (*N* = 187).

Demographic profile	Number of respondents	Percentage
Age		
56–60	130	69.52
61–65	37	19.79
66–70	15	8.02
71≥	5	2.67
Gender		
Male	119	63.64
Female	68	36.36
Educational background		
Junior High School or below	53	28.34
High School	61	32.62
College (Associate's Degree)	51	27.27
University (Bachelor's Degree)	19	10.16
Graduate School and above	3	1.6
Have you used mobile health systems before?		
Frequently	56	29.95
Occasionally	110	58.82
Never	27	11.23

**Figure 2:**
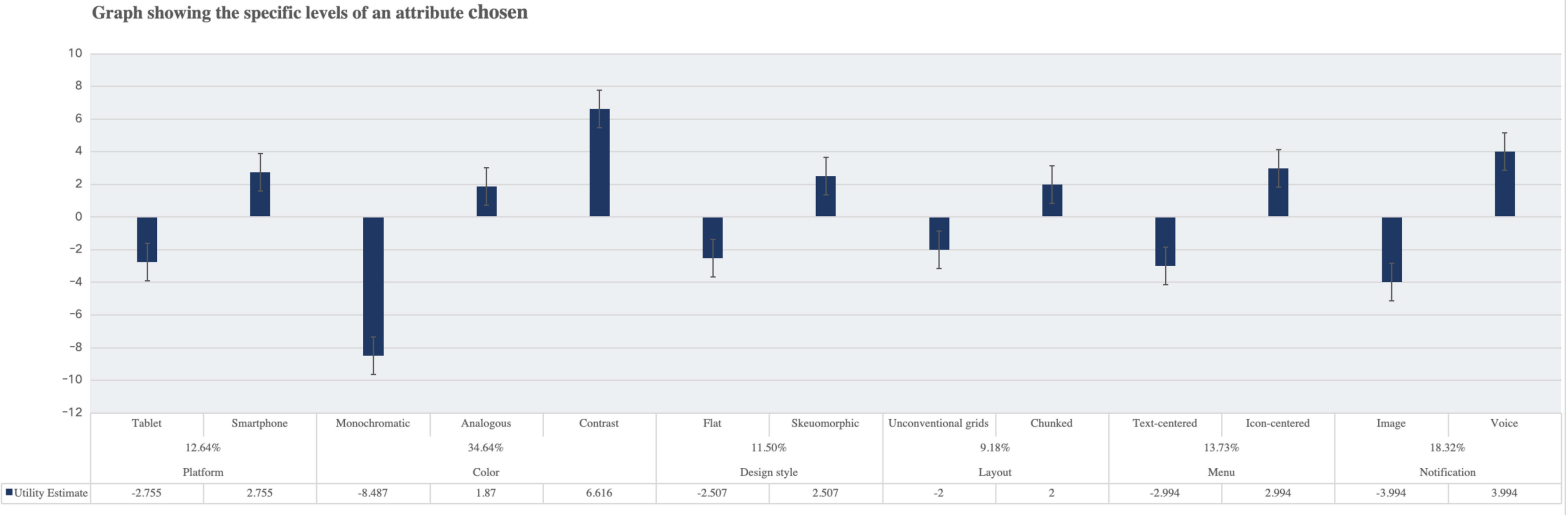
Graph showing the specific levels of an attribute.

The correlation of design attributes is elucidated in Table 4, where Pearson's R boasts a value of 0.921, accompanied by Kendall's Tau at 0.832. These values, hovering close to 1, unequivocally signify a potent relationship between the observed and estimated preferences (88). Emerging findings the elevated stature of certain attributes within the ePHR interface design landscape. Notably, the color scheme prominence, capturing a commanding 34.64% preference, illustrating its criticality amongst the elderly populace. This is subsequently trailed by the manner of message notifications, which garners a considerable 18.32% emphasis, Design style (11.50%) and layout formatting (9.18%) were considered to be of lower importance, as shown in Table 5. In Figure 2, the proportionate analysis of platform attributes explains the preference of elderly respondents for smaller-sized smartphone devices, i.e., the smaller and more portable the platform, the higher the proportion. Electronic health records featuring skeuomorphic design (*r* = 2.507), contrastive color schemes (*r* = 6.616), and grid-based chunk layout (*r* = 2) had the highest proportions. Preferences among the elderly for navigation menus centered around icons (*r* = 2.994) were greater than text, and they also favored receiving notification alerts via voice. The study reveals that older adults prefer mobile electronic health records with simpler layouts, dominated by icons, and featuring contrasting tones.

**Table 4:** Correlation.

	Value	Significance
Pearson's R	0.921	0.000
Kendall's Tau	0.832	0.000

**Table 5:** Preferences of healthcare providers on the ePHR interface.

Attribute	Importance value	Levels	Utility estimate
Platform	12.64%	Tablet	−2.755
		Smartphone	2.755
Color	34.64%	Monochromatic	−8.487
		Analogous	1.87
		Contrast	6.616
Design style	11.50%	Flat	−2.507
		Skeuomorphic	2.507
Layout	9.18%	Unconventional grids	−2
		Chunked	2
Menu Navigation	13.73%	Text-centered	−2.994
		Icon-centered	2.994
Notification	18.32%	Image	−3.994
		Voice	3.994

## 5 Discussion

The analysis unveils a pivotal insight, highlighting the color scheme as the paramount attribute affecting elderly users' interaction with the ePHR interface components. This discovery resonates coherently with existing research conducted by Hsieh, T.J (89). Which accentuates the instrumental role of colors in cultivating visual appeal, an aspect imperative for seizing and sustaining users' attention. Visual attributes like color choices are pivotal in enhancing the user experience, emphasizing the significance of visual processing in computer interaction. Such attributes are essential as they facilitate the capture of primary information from the external environment through vision. Numerous studies have corroborated that color is a principal visual feature affecting consumer cognition and behavior (90, 91).

Our findings suggest a proclivity among the elderly for smartphones as the primary device for ePHR access, attributed to their convenience and likely familiarity. Although tablets offer larger display sizes, they are often perceived as less portable and not integral for daily use. The key factors for this demographic appear to be portability and convenience, supporting the emerging trend toward lightweight device technologies (71). Undeniably, the choice of ePHR device platforms is a key factor in revealing user preferences for an ideal ePHR; users should be able to easily access digital health information across their choice of multiple device platforms.

Moreover, the study indicates that elderly users prefer color combinations with robust contrast. Considering visual impairments and age-related vision degradation, employing high-contrast color schemes enhances readability and interface discernibility, thereby improving the user experience. A homogenous color scheme can sometimes lead to recognition confusion, suggesting the need for some level of differentiation in interface colors for the elderly (92).

Thirdly, skeuomorphic design is favored over minimalism. Skeuomorphic design tends to imitate objects and textures from the real world, which may be easier for the elderly, who are less familiar with digital interfaces, to understand and use. For instance, an icon that looks like a real button or switch might make it more intuitive for them to comprehend how to operate it. This inclination is based on a widely accepted notion that skeuomorphic design in interfaces can accelerate recognition speed among users, a finding consistent with the research conducted by Cho, Minji et al. (60). This implies that ePHR designers should carefully consider incorporating visual symbols that closely resemble real-world objects when designing complex systems for elderly users. Such a practice facilitates understanding and reduces the likelihood of errors if the design is not user-friendly. Icons are significant graphical elements in interfaces with communication potential, reflecting not only the design style but also affecting interaction quality and user experience (58), ePHR designers should thus contemplate the use of visual symbols that closely resemble real-world objects to ease comprehension for the elderly, and if not designed appropriately, it could lead to an increase in user errors. Icons should be designed with clarity and should incorporate universally recognized symbols to minimize menu selection errors, thus avoiding potential complications in the information system. Engaging elderly users in participatory icon design is crucial to ensure clear communication and understanding of information and to implement consistency check procedures.

Fourthly, elderly users prefer interface layouts consisting of block-grid arrangements. This involves subdividing information into logically ordered sections. Researchers like Stone et al. have presented examples of interface layouts that might influence user usability (93) segregating substantial content into smaller visual blocks within the interface. Due to the increase in information quantity, centralized and grid layouts, where information is categorized into blocks, make usage and management easier. Such layouts also support the “pattern navigation” commonly displayed in the medical field (94), enabling quick browsing through multiple pieces of information to find necessary data for diagnosis or treatment plans. A well-structured interface minimizes cognitive load, allowing users to easily locate required content without feeling overwhelmed. Block-grid layouts are not merely an aesthetic choice; they are a user-centric option reflecting the tool's application in the actual workflow of healthcare delivery. This aligns with the importance of user interface awareness as mentioned by Shneiderman, benefiting any domain that necessitates the management of complex screen information. Such designs contribute to efficient and effective usage.

Fifthly, the elderly population in this study indicated a stronger preference for “icon-centric” over “text-centric” navigation menus. Icons more effectively capture visual interest, addressing potential difficulties elderly users may face when reading or understanding extensive text information. Employing an icon-centric navigation menu enables more intuitive information conveyance, making it easier for users to locate the desired options and features. Stone et al. and colleagues have identified various interface layout examples that could impact user usability (93), Segmenting a large amount of content into smaller visual blocks within the interface facilitates easier management, especially as the volume of information increases. This approach aligns with findings by Schneiderman and Pleasant (23), that graphical interfaces are preferable to text-based ones, as they reduce the time cost of reading by substituting appropriate icons for extended text.

Finally, it is noteworthy to emphasize the preferences of elderly users concerning feedback mechanisms in ePHR. Auditory feedback is particularly favored by this demographic. Given the age-related deterioration of vision, the limitations of screen-based visual cues may result in less effective feedback compared to the immediacy and directness of auditory cues. Additionally, audio feedback allows them to engage with the application or features without excessive dispersion of their attention. This is also applicable for those who may not be comfortable reading on-screen text or easily comprehending graphical interfaces. Therefore, the system design should incorporate auditory information feedback. This corroborates the findings that human-computer interaction design should incorporate multi-sensory interaction pathways to improve the user experience (40), As the interface is the critical medium for elderly users to interact with intelligent devices, considerations should be given to their physiological characteristics, cognitive habits, psychological inclinations, and usage preferences.

While this research boasts compelling findings and outcomes, there remain some limitations. Due to the subjective evaluation statistical method employed, the number of attributes in the conjoint analysis typically does not exceed six, implying that not all facets of interactive interface design are fully addressed. Additionally, the limited sample size primarily consists of elderly individuals residing in Chinese cities and obtained through online surveys. This sample does not include the elderly with severe illnesses or those facing difficulties with mobile device technology. Given the potential variations in preferences among elderly populations with different mobility capabilities and backgrounds, there might be biases in the design preferences identified. In future endeavors, we aim to delve deeper by segmenting the elderly based on their abilities, covering a broader spectrum including nursing homes. Moreover, usability experiments related to system development will be carried out.

## 6 Conclusion

Driven by the need to improve healthcare accessibility, it is essential to promote the development of a health-conscious aging society by designing digital system products tailored to the needs of the elderly. This is particularly pertinent within the context of emerging mobile healthcare paradigms. To achieve digital accessibility, it is crucial to take into account the unique characteristics and habits of elderly users during the design process.

Our research outcomes shed light on the focal points of elderly users' attention towards ePHR interface design attributes and their preferences for design elements within user interfaces. Through relative comparisons of combined elements, the conjoint analysis methodology is able to accurately capture users’ evaluations of interface attribute factors. This offers a more objective and precise perspective compared to individual assessments. Specifically, designs incorporating contrasting colors, grid-based layouts, skeuomorphic design, and icon-centric menu navigation were identified as the most practical components. This underscores the significance of visual cognition processes in enhancing ePHR usability. Engaging the elderly, a distinct demographic, in surveys not only offers them a pleasant experience but also yields constructive outcomes. These insights provide valuable reference points for the design and development process of ePHR systems for elderly users, aiming to match their needs and preferences, thereby elevating their overall satisfaction and accomplishing the goal of enhanced system usability. Furthermore, our approach of dissecting constitutive elements and combining scenarios for studying interactive interface design can be extended and further researched in other health product domains. Although the aging population presents a series of challenges to society and healthcare systems, there is an incentive to construct ePHR as a health information management tool or application. This promotes self-health management and information sharing among the elderly. By planning, innovating, and collaborating, stakeholders beyond healthcare professionals can better support the health and well-being of the elderly, enhance the dissemination of geriatric healthcare knowledge, and boost electronic health literacy. All these initiatives actively empower the progression of an aging society.

## Data Availability

The original contributions presented in the study are included in the article/Supplementary Material, further inquiries can be directed to the corresponding author.

## References

[1] Ortman JM Velkoff VA Hogan H. An Aging Nation: the Older Population in the United States (2014).

[2] He W Goodkind D Kowal PR. An Aging World: 2015. Washington, DC: United States Census Bureau (2016).

[3] Chen X Giles J Yao Y Yip W Meng Q Berkman L The path to healthy ageing in China: a peking university–lancet commission. Lancet. (2022) 400:1967–2006. 10.1016/S0140-6736(22)01546-X 36423650 PMC9801271

[4] Pinto JM Fontaine AM Neri AL. The influence of physical and mental health on life satisfaction is mediated by self-rated health: a study with Brazilian elderly. Arch Gerontol Geriatr. (2016) 65:104–10. 10.1016/j.archger.2016.03.009 27017415

[5] Pian W Song S Zhang Y. Consumer health information needs: a systematic review of measures. Inf Process Manag. (2020) 57:102077. 10.1016/j.ipm.2019.102077

[6] Medlock S Eslami S Askari M Arts DL Sent D De Rooij SE Health information–seeking behavior of seniors who use the internet: a survey. J Med Internet Res. (2015) 17:e10. 10.2196/jmir.3749 25574815 PMC4296102

[7] Czaja SJ. Long-term care services and support systems for older adults: the role of technology. Am Psychol. (2016) 71:294. 10.1037/a0040258 27159436

[8] Heart T Ben-Assuli O Shabtai I. A review of PHR, EMR and EHR integration: a more personalized healthcare and public health policy. Health Policy Technol. (2017) 6:20–5. 10.1016/j.hlpt.2016.08.002

[9] Adetoyi OE Raji OA. Electronic health record design for inclusion in sub-saharan Africa medical record informatics. Sci Afr. (2020) 7:e00304. 10.1016/j.sciaf.2020.e00304

[10] Kukafka R Ancker JS Chan C Chelico J Khan S Mortoti S Redesigning electronic health record systems to support public health. J Biomed Inform. (2007) 40:398–409. 10.1016/j.jbi.2007.07.001 17632039

[11] Highfill T. Do hospitals with electronic health records have lower costs? A systematic review and meta-analysis. Int J Healthc Manag. (2020) 13(1):65–71. 10.1080/20479700.2019.1616895

[12] Saleem JJ Russ AL Neddo A Blades PT Doebbeling BN Foresman BH. Paper persistence, workarounds, and communication breakdowns in computerized consultation management. Int J Med Inf. (2011) 80:466–79. 10.1016/j.ijmedinf.2011.03.016 21530383

[13] Archer N Fevrier-Thomas U Lokker C Mckibbon KA Straus SE. Personal health records: a scoping review. J Am Med Inform Assoc. (2011) 18:515–22. 10.1136/amiajnl-2011-000105 21672914 PMC3128401

[14] Johnson J. Designing with the Mind in Mind: Simple Guide to Understanding User Interface Design Guidelines. Amsterdam: Elsevier (2020).

[15] Tanioka R Yasuhara Y Osaka K Kai Y Zhao Y Tanioka T Autonomic nervous activity of patient with schizophrenia during pepper CPGE-led upper limb range of motion exercises. Enferm Clin. (2020) 30:48–53. 10.1016/j.enfcli.2019.09.023

[16] Yasuhara Y Tanioka T Kai Y Tsujigami Y Uematsu K Dino MJS Potential legal issues when caring healthcare robot with communication in caring functions are used for older adult care. Enferm Clin. (2020) 30:54–9. 10.1016/j.enfcli.2019.09.024

[17] Yun Y Ma D Yang M. Human–computer interaction-based decision support system with applications in data mining. Future Gener Comput Syst. (2021) 114:285–9. 10.1016/j.future.2020.07.048

[18] Nishitani H. Human-computer interaction in the new process technology. J Process Control. (1996) 6:111–7. 10.1016/0959-1524(96)86053-7

[19] Hinss MF Brock AM Roy RN. Cognitive effects of prolonged continuous human-machine interaction: the case for mental state-based adaptive interfaces. Front Neuroergonomics. (2022) 3:935092. 10.3389/fnrgo.2022.935092 PMC10790890 38235472

[20] Rahimi B Vimarlund V Timpka T. Health information system implementation: a qualitative meta-analysis. J Med Syst. (2009) 33:359–68. 10.1007/s10916-008-9198-9 19827262

[21] Gooch P Roudsari A. Computerization of workflows, guidelines, and care pathways: a review of implementation challenges for process-oriented health information systems. J Am Med Inform Assoc. (2011) 18:738–48. 10.1136/amiajnl-2010-000033 21724740 PMC3197986

[22] Jilka SR Callahan R Sevdalis N Mayer EK Darzi A. “Nothing about me without me”: an interpretative review of patient accessible electronic health records. J Med Internet Res. (2015) 17:e161. 10.2196/jmir.4446 26123476 PMC4526966

[23] Shneiderman B Plaisant C Cohen MS Jacobs S Elmqvist N Diakopoulos N. Designing the User Interface: Strategies for Effective Human-Computer Interaction. Chennai: Pearson Education India (2010).

[24] Yang J Ouyang W Chen Y. Research on human-machine interface design of smart refrigerator based on conjoint analysis. J Phys. (2021) 2029(1):012046. 10.1088/1742-6596/2029/1/012046

[25] Pleger LE Mertes A Rey A Brüesch C. Allowing users to pick and choose: a conjoint analysis of end-user preferences of public e-services. Gov Inf Q. (2020) 37:101473. 10.1016/j.giq.2020.101473

[26] Igarashi A Nakano Y Yoneyama-Hirozane M. Public preferences and willingness to accept a hypothetical vaccine to prevent a pandemic in Japan: a conjoint analysis. Expert Rev Vaccines. (2022) 21:241–8. 10.1080/14760584.2022.2016402 35073824

[27] Wildenbos GA Horenberg F Jaspers M Peute L Sent D. How do patients value and prioritize patient portal functionalities and usage factors? A conjoint analysis study with chronically ill patients. BMC Med Inform Decis Mak. (2018) 18:1–10. 10.1186/s12911-018-0708-5 30463613 PMC6249922

[28] Yuan T Guo Y. Gamification design of health apps for the elderly based on the kano model and conjoint analysis method. International Conference on Human-Computer Interaction (2021). p. 176–90, Springer.

[29] O’Caoimh R Molloy DW Fitzgerald C Van Velsen L Cabrita M Nassabi MH ICT-supported interventions targeting pre-frailty: healthcare recommendations from the personalised ICT supported service for independent living and active ageing (PERSSILAA) study. Information and Communication Technologies for Ageing Well and E-Health: Third International Conference, ICT4AWE 2017; April 28–29, 2017; Porto, Portugal (2018), Springer. p. 69–92, Revised Selected Papers 3.

[30] Brennan PF Downs S Casper G. Project HealthDesign: rethinking the power and potential of personal health records. J Biomed Inform. (2010) 43:S3–5. 10.1016/j.jbi.2010.09.001 20937482

[31] Wynia M Dunn K. Dreams and nightmares: practical and ethical issues for patients and physicians using personal health records. J Law Med Ethics. (2010) 38:64–73. 10.1111/j.1748-720X.2010.00467.x 20446985

[32] Tang PC Ash JS Bates DW Overhage JM Sands DZ. Personal health records: definitions, benefits, and strategies for overcoming barriers to adoption. J Am Med Inform Assoc. (2006) 13:121–6. 10.1197/jamia.M2025 16357345 PMC1447551

[33] Kettley P Reilly P. eHR: An Introduction. IES Report, ERIC (2003).

[34] Pagliari C Detmer D Singleton P. Potential of electronic personal health records. Br Med J. (2007) 335:330–3. 10.1136/bmj.39279.482963.AD 17703042 PMC1949437

[35] Kaelber D Pan EC. The value of personal health record (PHR) systems. AMIA Annu Symp Proc. (2008) 2008:343–7. PMID: 18999276; PMCID: PMC2655982. 18999276 PMC2655982

[36] Spil T Klein R. The personal health future. Health Policy Technol. (2015) 4:131–6. 10.1016/j.hlpt.2015.02.004

[37] Czaja SJ Zarcadoolas C Vaughon WL Lee CC Rockoff ML Levy J. The usability of electronic personal health record systems for an underserved adult population. Hum Factors. (2015) 57:491–506. 10.1177/0018720814549238 25875437 PMC4710573

[38] Price MM Pak R Müller H Stronge A. Older adults’ perceptions of usefulness of personal health records. Univers Access Inf Soc. (2013) 12:191–204. 10.1007/s10209-012-0275-y

[39] Hunsaker A Hargittai E. A review of internet use among older adults. New Media Soc. (2018) 20:3937–54. 10.1177/1461444818787348

[40] Bernardo J Apóstolo J Loureiro R Santana E Yaylagul NK Dantas C Ehealth platforms to promote autonomous life and active aging: a scoping review. Int J Environ Res Public Health. (2022) 19:15940. 10.3390/ijerph192315940 36498018 PMC9738367

[41] Pirhonen J Lolich L Tuominen K Jolanki O Timonen V. “These devices have not been made for older people’s needs”–older adults’ perceptions of digital technologies in Finland and Ireland. Technol Soc. (2020) 62:101287. 10.1016/j.techsoc.2020.101287

[42] Ford EW Hesse BW Huerta TR. Personal health record use in the United States: forecasting future adoption levels. J Med Internet Res. (2016) 18:e73. 10.2196/jmir.4973 27030105 PMC4830902

[43] Andrikopoulou E Scott P Herrera H Good A. What are the important design features of personal health records to improve medication adherence for patients with long-term conditions? A systematic literature review. BMJ Open. (2019) 9:e028628. 10.1136/bmjopen-2018-028628 31558449 PMC6773318

[44] Park HS Cho H Kim HS. Development of a multi-agent m-health application based on various protocols for chronic disease self-management. J Med Syst. (2016) 40:1–14. 10.1007/s10916-015-0365-5 26573657

[45] Coleman A. Medication adherence of elderly citizens in retirement homes through a mobile phone adherence monitoring framework (Mpamf) for developing countries: a case study in South Africa. Indian J Pharm Educ Res. (2014) 48:6–11. 10.5530/ijper.48.3.2

[46] Tseng M-H Wu H-C. A cloud medication safety support system using QR code and web services for elderly outpatients. Technol Health Care. (2014) 22:99–113. 10.3233/THC-140778 24561883

[47] Price M Bellwood P Kitson N Davies I Weber J Lau F. Conditions potentially sensitive to a personal health record (PHR) intervention, a systematic review. BMC Med Inform Decis Mak. (2015) 15:1–12. 10.1186/s12911-015-0159-1 25927384 PMC4411701

[48] Grudin J. From tool to partner: The evolution of human-computer interaction. Synth Lect Hum-Cent Interact. (2017) 10(1):i-183.

[49] Kim H Chen J Kim E Agogino AM. Scenario-based conjoint analysis: measuring preferences for user experiences in early stage design. International Design Engineering Technical Conferences and Computers and Information in Engineering Conference (2017). American Society of Mechanical Engineers, V007T06A042.

[50] Ludwin S Greysen SR. Use of smartphones and mobile devices in hospitalized patients: untapped opportunities for inpatient engagement. J Hosp Med. (2015) 10:459. 10.1002/jhm.2365 25872902 PMC4490991

[51] Salehi HP. Smartphone for healthcare communication. J Healthcare Commun. (2018) 3:34. 10.4172/2472-1654.100144

[52] Ryu B Kim N Heo E Yoo S Lee K Hwang H Impact of an electronic health record-integrated personal health record on patient participation in health care: development and randomized controlled trial of MyHealthKeeper. J Med Internet Res. (2017) 19:e401. 10.2196/jmir.8867 29217503 PMC5740264

[53] Baym NK. Personal Connections in the Digital Age. Cambridge, UK: Polity Press (2010).

[54] Kim S Yao W Du X. Exploring older adults’ adoption and use of a tablet computer during COVID-19: longitudinal qualitative study. JMIR Aging. (2022) 5:e32957. 10.2196/32957 35134747 PMC8906838

[55] Palmer SE Schloss KB. An ecological valence theory of human color preference. Proc Natl Acad Sci USA. (2010) 107:8877–82. 10.1073/pnas.0906172107 20421475 PMC2889342

[56] Bischof D. New graphic schemes for stata: plotplain and plottig. Stata J. (2017) 17:748–59. 10.1177/1536867X1701700313

[57] Koshman S. Testing user interaction with a prototype visualization-based information retrieval system. J Am Soc Inf Sci Technol. (2005) 56:824–33. 10.1002/asi.20175

[58] Salman YB Cheng H-I Kim JY Patterson PE. Medical information system with iconic user interfaces. Int J Digit Content Technol Appl. (2010) 4:137–48. 10.4156/jdcta.vol4.issue1.14

[59] Hekkert P. Design aesthetics: principles of pleasure in design. Psychol Sci. (2006) 48:157.

[60] Cho M Kwon S Na N Suk H-J Lee K. The elders preference for skeuomorphism as app icon style. Proceedings of the 33rd Annual ACM Conference Extended Abstracts on Human Factors in Computing Systems (2015). p. 899–904

[61] Farzandipour M Nabovati E Farrokhian A Akbari H Sharif R. Designing and usability assessing an electronic personal health record for patients with chronic heart failure in a developing country. Inform Med Unlocked. (2021) 27:100804. 10.1016/j.imu.2021.100804

[62] Blåsjö M Johansson S Jonsson C. “Put a meeting in my calendar!” the literacy practice of the digital calendar in workplaces. Sakprosa. (2019) 11(1):1–47. 10.5617/sakprosa.5951

[63] Wiedenbeck S. The use of icons and labels in an end user application program: an empirical study of learning and retention. Behav Inf Technol. (1999) 18:68–82. 10.1080/014492999119129

[64] Sänn A Baier D. Lead user identification in conjoint analysis based product design. Challenges at the Interface of Data Analysis, Computer Science, and Optimization: Proceedings of the 34th Annual Conference of the Gesellschaft Für Klassifikation e. V.; July 21–23, 2010; Karlsruhe (2012). p. 521–8, Springer.

[65] Luce RD Tukey JW. Simultaneous conjoint measurement: a new type of fundamental measurement. J Math Psychol. (1964) 1:1–27. 10.1016/0022-2496(64)90015-X

[66] Rao VR. Conjoint analysis. In: Sheth J Malhotra N, editors. Wiley International Encyclopedia of Marketing. Chichester: John Wiley & Sons, Ltd (2010). 10.1002/9781444316568.wiem02019

[67] Li W Long R Chen H Dou B Chen F Zheng X Public preference for electric vehicle incentive policies in China: a conjoint analysis. Int J Environ Res Public Health. (2020) 17:318. 10.3390/ijerph17010318 31906526 PMC6981758

[68] Zurovac J Brown R. Orthogonal Design: A Powerful Method for Comparative Effectiveness Research with Multiple Interventions. Issue Brief. Washington, DC: Center on Health Care Effectiveness (2012).

[69] Min SH Kim HY Kwon YJ Sohn SY. Conjoint analysis for improving the e-book reader in the Korean market. Expert Syst Appl. (2011) 38:12923–9. 10.1016/j.eswa.2011.04.087

[70] Kim JH Choi WS Song JY Yoon YK Kim MJ Sohn JW. The role of smart monitoring digital health care system based on smartphone application and personal health record platform for patients diagnosed with coronavirus disease 2019. BMC Infect Dis. (2021) 21:1–8. 10.1186/s12879-020-05706-z 33639861 PMC7910795

[71] Alhur A. Exploring Saudi Arabia Individuals’ attitudes toward electronic personal health records. J Comput Sci Technol Studies. (2022) 4:80–7. 10.32996/jcsts.2022.4.1.10

[72] Greysen SR Khanna RR Jacolbia R Lee HM Auerbach AD. Tablet computers for hospitalized patients: a pilot study to improve inpatient engagement. J Hosp Med. (2014) 9:396–9. 10.1002/jhm.2169 24523051 PMC4043916

[73] Camgöz N Yener C Güvenç D. Effects of hue, saturation, and brightness on preference. Color Res Appl. (2002) 27:199–207. 10.1002/col.10051

[74] Chrysos G Dollas A Bourbakis N. An embedded software-reconfigurable color segmentation architecture for image processing systems. Microprocess Microsyst. (2012) 36:215–31. 10.1016/j.micpro.2011.12.004

[75] Cyr D Head M Larios H. Colour appeal in website design within and across cultures: a multi-method evaluation. Int J Hum Comput Stud. (2010) 68:1–21. 10.1016/j.ijhcs.2009.08.005

[76] Bonnardel N Piolat A Le Bigot L. The impact of colour on website appeal and users’ cognitive processes. Displays. (2011) 32:69–80. 10.1016/j.displa.2010.12.002

[77] Chapman C. Color Theory for Designer, Part 3: Creating Your Own Color Palettes (2010).

[78] Page T. Skeuomorphism or flat design: future directions in mobile device User Interface (UI) design education. Int J Mobile Learn Organ. (2014) 8:130–42. 10.1504/IJMLO.2014.062350

[79] Spiliotopoulos K Rigou M Sirmakessis S. A comparative study of skeuomorphic and flat design from a UX perspective. Multimodal Technol Interaction. (2018) 2:31. 10.3390/mti2020031

[80] Lauffenburger JC Isaac T Trippa L Keller P Robertson T Glynn RJ Rationale and design of the novel uses of adaptive designs to guide provider engagement in electronic health records (NUDGE-EHR) pragmatic adaptive randomized trial: a trial protocol. Implement Sci. (2021) 16:1–11. 10.1186/s13012-020-01078-9 33413494 PMC7792313

[81] Rouse M. Search Mobile Computing. [línea]. Available Online at: Available at: https://searchmobilecomputing.techtarget.com/definition/GPRS [Último acceso: 13 Enero 2019] (2007).

[82] Sharit J Lisigurski M Andrade AD Karanam C Nazi KM Lewis JR The roles of health literacy, numeracy, and graph literacy on the usability of the VA’s personal health record by veterans. J Usability Stud. (2014) 9:173–93.

[83] Cuddihy E Spyridakis JH. The effect of visual design and placement of intra-article navigation schemes on Reading comprehension and website user perceptions. Comput Human Behav. (2012) 28:1399–409. 10.1016/j.chb.2012.03.002

[84] Salman YB Cheng H-I Patterson PE. Icon and user interface design for emergency medical information systems: a case study. Int J Med Inf. (2012) 81:29–35. 10.1016/j.ijmedinf.2011.08.005 21920810

[85] Jin X Hu X Wei X Fan M. Synapse: interactive guidance by demonstration with trial-and-error support for older adults to use smartphone apps. Proc ACM Interact Mob Wearable Ubiquitous Technol. (2022) 6:1–24. 10.1145/3550321

[86] Hartson R. Cognitive, physical, sensory, and functional affordances in interaction design. Behav Inf Technol. (2003) 22:315–38. 10.1080/01449290310001592587

[87] Wohllebe A Adler MR Podruzsik S. Influence of design elements of mobile push notifications on mobile app user interactions. Int J Interact Mob Technol. (2021) 15:35–46. 10.3991/ijim.v15i15.23897

[88] Chok NS. Pearson’s versus Spearman’s and Kendall’s Correlation Coefficients for Continuous Data. Doctoral dissertation. Pittsburgh: University of Pittsburgh (2010).

[89] Hsieh TJ. Multiple roles of color information in the perception of icon-type images. Color Res Appl. (2017) 42:740–52. 10.1002/col.22140

[90] Kauppinen-Räisänen H Jauffret M-N. Using colour semiotics to explore colour meanings. Qual Market Res. (2018) 21:101–17. 10.1108/QMR-03-2016-0033

[91] Aslam MM. Are you selling the right colour? A cross-cultural review of colour as a marketing cue. J Market Commun. (2006) 12:15–30. 10.1080/13527260500247827

[92] Seymour KJ Williams MA Rich AN. The representation of color across the human visual cortex: distinguishing chromatic signals contributing to object form versus surface color. Cerebral Cortex. (2016) 26:1997–2005. 10.1093/cercor/bhv021 25681421

[93] Stone D Jarrett C Woodroffe M Minocha S. User Interface Design and Evaluation. Morgan Kaufmann Series in Interactive Technologies. San Francisco: Morgan Kaufman (2005).

[94] Thyvalikakath TP Dziabiak MP Johnson R Torres-Urquidy MH Acharya A Yabes J Advancing cognitive engineering methods to support user interface design for electronic health records. Int J Med Inf. (2014) 83:292–302. 10.1016/j.ijmedinf.2014.01.007 PMC3977320 24503391

